# Late Recurrence of Benign Multicystic Peritoneal Mesothelioma Complicated with an Incisional Hernia

**DOI:** 10.1155/2013/903795

**Published:** 2013-03-07

**Authors:** Emel Canbay, Haruaki Ishibashi, Shouzou Sako, Toshiyuki Kitai, Eisei Nishino, Yutaka Yonemura

**Affiliations:** ^1^NPO to Support Peritoneal Dissemination Treatment, 1-26, Harukimotomachi, Kishiwada City, Osaka 596-0032, Japan; ^2^Department of General Surgery, Kishiwada Tokushukai Hospital, 4-27-1 Kamori-Cho, Kishiwada City, Osaka 596-8522, Japan; ^3^Department of General Surgery, Kocaeli-Derince Education and Research Hospital, Kocaeli 41900, Turkey; ^4^Department of Pathology, Kishiwada Tokushukai Hospital, 4-27-1 Kamori-Cho, Kishiwada City, Osaka 596-8522, Japan

## Abstract

Benign multicystic peritoneal mesothelioma (BMPM) is a rare disease arising from the peritoneal mesothelium. Here, we report a 57-year-old woman admitted to our unit with an incisional hernia fifteen years later following her first operation due to BMPM. Computerized tomography demonstrated a cystic appearing mass with intraabdominal extension in hernia sac. The patient underwent en bloc resection of the mass and hernia repair. An immunohistochemical analysis of the mass confirmed the recurrence of BMPM. Our case supports that BMPM has slowly progressive nature and can recur with complicated incisional hernia long time after primary resection. Diagnosis and long-term followup are crucial for clarifying the characteristics of this disease.

## 1. Introduction

Benign multicystic peritoneal mesothelioma (BMPM; peritoneal inclusion cyst, multilocular inclusion cyst, and benign multicystic mesothelioma) is a very rare multilocular cystic tumor arises from the peritoneal mesothelium [1, 2]. The etiology and pathogenesis are controversial regarding its neoplastic and reactive nature. The majority of these patients present with chronic or intermittent lower abdominal or pelvic pain. However, cases with retroperitoneal mass [3] or acute abdomen [4] have also been described. The diagnosis of BMPM is confirmed by electron microscopy and immunohistochemistry. Although BMPM is associated with a favorable short-term prognosis, there is increased recurrence during long-term followup [2].

We herein describe the late recurrence of BMPM complicated with incisional hernia fifteen years later following her first abdominal operation due to BMPM.

## 2. Case Presentation

**Figure 1:**
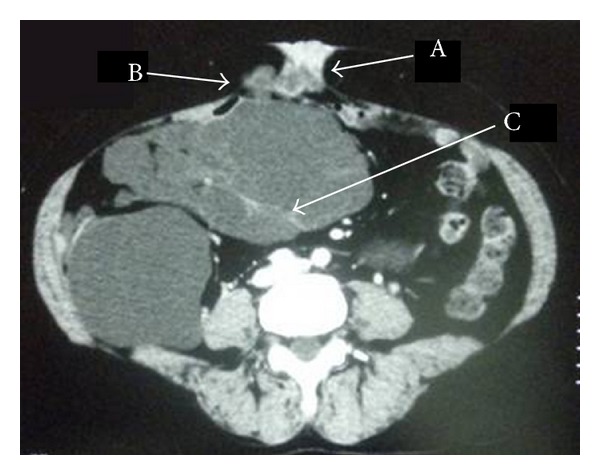
Computed tomography images of the BMPM: the arrows indicate the incisional hernia (A), multicystic mass surrounded to umbilicus (B), and intraabdominal extension (C).

A 57-year-old woman presented to our unit with an incisional hernia due to enlarged abdominal mass surrounded to the umbilicus fifteen years later following her first abdominal operation. She had a past medical history of BMPM originated from mesentery of appendix with extension to the ascending colon and she underwent right hemicolectomy. Since then, she was followed up four times a year in first two years then a year basis after 2 years of her operation. She did not go annual followup after 10 years. She reported abdominal discomfort and distension fifteen years later following her first operation. She attributed this to her incisional hernia at the site of midline incision. These had progressively worsened in size as had her symptoms. Her physical examination revealed painful palpable abdominal mass about 4 cm in size around the umbilicus and incisional hernia that lays 10 mm above and below her umbilicus and 30 mm diameter. Computed tomography (CT) examination demonstrated an intraperitoneal hypodense cystic mass surrounded to umbilicus and extended between the great curvature of the stomach, spleen, and tail of pancreas and extending caudally to the upper margin of the pelvis and pressure over the adjacent organs resulted with hernia from midline incision scar (Figure 1). She underwent operation and multicystic mass was found to herniated and surrounded to umbilicus. A complete resection of the mass and the umbilicus and hernia repairment were performed. She had an uneventful postoperative recovery.

**Figure 2:**
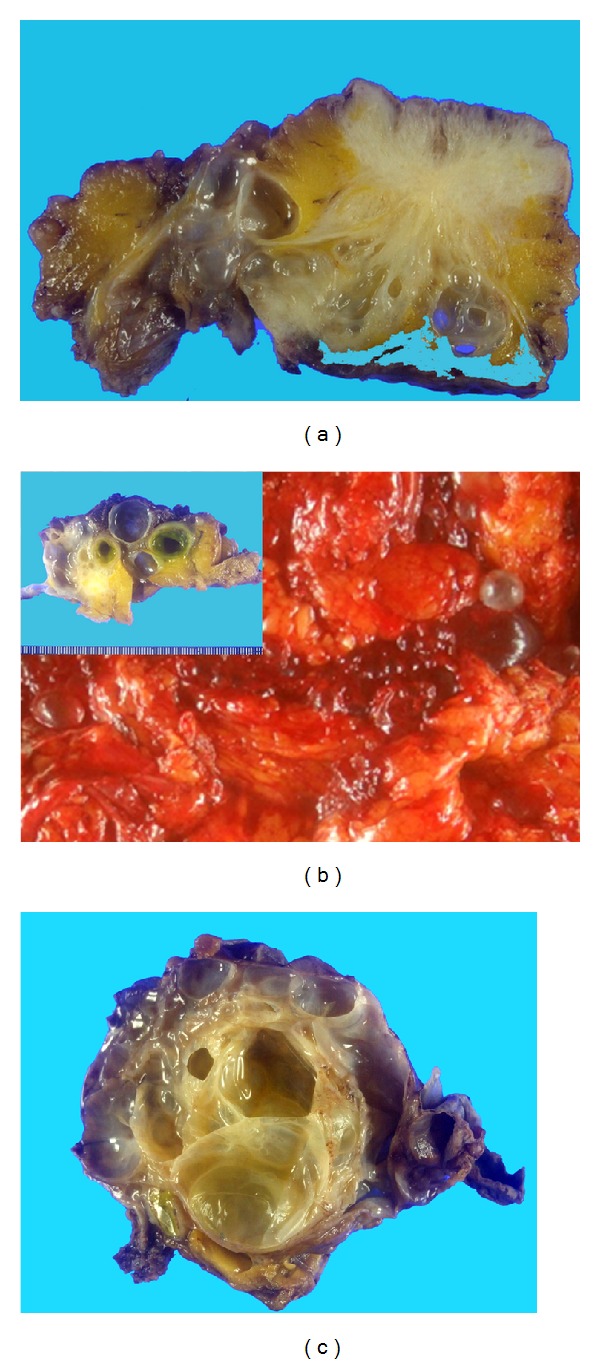
The multicystic mass surrounded to umbilicus (a), greater omentum (b), and mesentery of small bowel (c).

**Figure 3:**
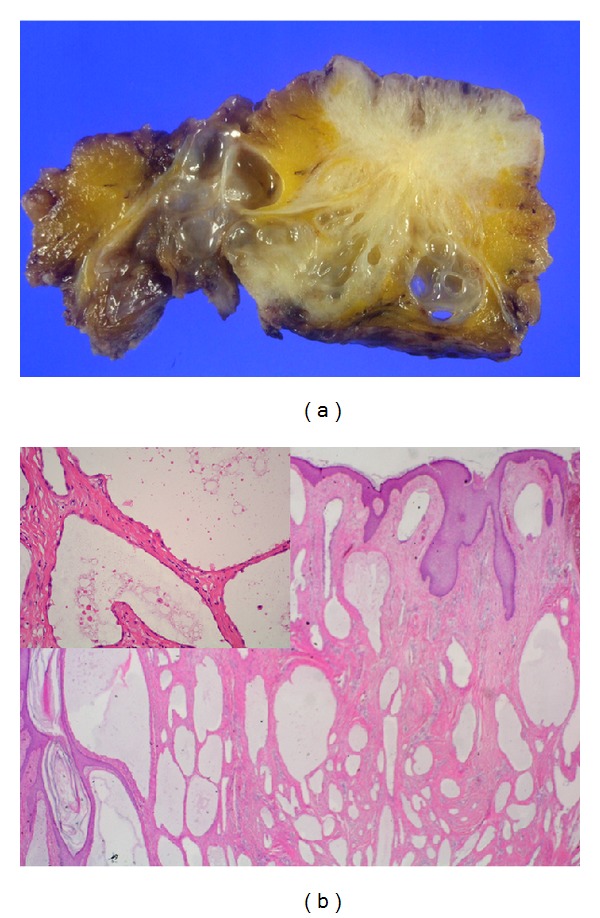
Multiple, thin walled irregular shaped cysts growing in the umbilicus (a), thin-walled cysts lined by flattened or cuboidal mesothelial cells (b).

**Figure 4:**
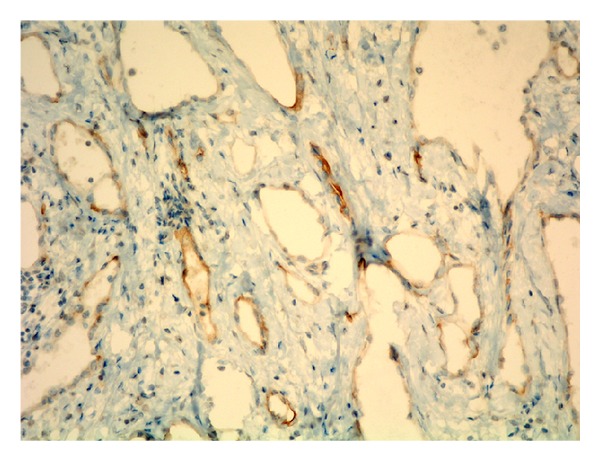
Calretinin positive cells surrounding cysts.

Macroscopic examination of large cystic mass containing multiple smaller cystic spaces was shown in Figure 2. Microscopic examination of the cysts revealed that the walls of the cysts were lined by single flat cells alternating with cuboidal cells with hobnail features (Figure 3). The nature of the lining cells was confirmed by immunohistochemical staining for cytokeratin 5/6 and calretinin (Figure 4) as a BMPM.

## 3. Discussion

Multicystic mesothelioma of the peritoneum was first described by Plaut in 1928, but the mesothelial nature was confirmed with electron microscopy by Mennemeyer and Smith in 1979 [5]. 

 The disease is an exceedingly rare medical entity, which renders its origin, pathogenesis, diagnosis, and therapy challenging [2, 4]. BMPM occurs predominantly in women child-bearing age and is associated with a history of previous abdominal surgery, endometriosis, leiomyomas, or pelvic inflammatory disease (PID) [2]. Only few reports exist of BMPM in elderly [4, 6, 7]. 

The pathogenesis of BMPM is unclear and there is some controvercy regarding its neoplastic or reactive nature [1, 2, 8]. Malignant transformation of BMPM as an unusual occurrence indicating a neoplastic nature underscoring the necessity of long-term followup has also been reported previously [8]. Evidence that BMPM represents a reactive process includes that it occurs in females with a history of endometriosis, prior abdominal surgery, or pelvic inflammatory disease [2, 9]. 

Most patients are diagnosed incidentally during physical examination or surgery for other reasons. The clinical findings of benign multicystic mesotheliomas are nonspecific and include nausea, vomiting, and abdominal pain. Safioleas et al. [4] also have been reported its presentation as an incisional incarcerated hernia. Our case was attended to our hospital with an incisional hernia, abdominal discomfort, and a mass surrounded to the umbilicus. This makes our case unique in terms of presentation that recurrence can be seen with incisional hernia due to enlarged volume of the tumor ended up with the expression over the intraabdominal viscera and herniation from the abdominal wall. Laboratory findings are nonspecific for diagnosis of BMPM. CT provides more information about the location and the extent of the mass demonstrates well-defined, low-attenuation mass with non-calcified septa [10]. An intraperitoneal hypodense cystic mass in surrounding umbilicus with an intraabdominal extension and pressure over the adjacent organs, resulted with incisional hernia, was demonstrated with CT in our case. 

Histologically, BMPM is a localized tumor arising from mesothelial cells. The tumor is composed of a multiple mesothelial-lined cystic structure. Surgery is the only effective treatment. Aggressive surgical approaches including cytoreductive surgery with peritonectomy are recommended [4, 11]. Complete cytoreductive surgery was achieved in our patient.

Prognosis of BMPM was reported differently. Weiss and Tavassoli reported that the prognosis was excellent and only death was occurred in a patient who refused the surgery [1]. Beside this, it has been reported that a woman at aged 36 with BMPM had developed a diffuse malignant mesothelioma after six surgical procedures [8]. Tumor recurrence was reported in half of the patients even complete cytoreduction was achieved [1, 12]. Therefore, routine followup with imaging techniques is required in patients after operation [13]. Our patient was ended up with a recurrence of BMPM 15 years later following her first operation suggesting long term followup is necessary in patients with BMPM. 

In conclusion, recognition of BMPM is difficult and it can be recur as an incisional hernia. Recurrence of disease and uncertainty of biological behaviors indicate the necessity of proper long-term followup of patients with BMPM. In addition to this, complete cytoreductive surgery seems to be the proper management of this disease. 

## References

[1] Weiss SW, Tavassoli FA (1988). Multicystic mesothelioma. An analysis of pathologic findings and biologic behavior in 37 cases. *American Journal of Surgical Pathology*.

[2] Ross MJ, Welch WR, Scully RE (1989). Multiocular peritoneal inclusion cysts (so-called cystic mesotheliomas). *Cancer*.

[3] Villaschi S, Autelitano F, Santeusanio G, Balistreri P (1990). Cystic mesothelioma of the peritoneum. A report of three cases. *American Journal of Clinical Pathology*.

[4] Safioleas MC, Constantinos K, Michael S, Konstantinos G, Constantinos S, Alkiviadis K (2006). Benign multicystic peritoneal mesothelioma: a case report and review of the literature. *World Journal of Gastroenterology*.

[5] Mennemeyer R, Smith M (1979). Multicystic, peritoneal mesothelioma. A report with electron microscopy of a case mimicking intra-abdominal cystic hygroma (lymphangioma). *Cancer*.

[6] Snyder JA, Carman R, Aggon AA, Cardinale JP (2011). Benign multicystic peritoneal mesothelioma: a rare case presenting as pneumoperitoneum and pneumotosis intestinalis. *Journal of Gastrointestinal Oncology*.

[7] Husain A, Ozdemirli M (2012). Benign multicystic mesothelioma with concurrent colonic adenocarcinoma: a report of two cases. *Surgery Today*.

[8] González-Moreno S, Yan H, Alcorn KW, Sugarbaker PH (2002). Malignant transformation of “Benign” cystic mesothelioma of the peritoneum. *Journal of Surgical Oncology*.

[9] Groisman GM, Kerner H (1992). Multicystic mesothelioma with endometriosis. *Acta Obstetricia et Gynecologica Scandinavica*.

[10] Pitta X, Andreadis E, Ekonomou A (2010). Benign multicystic peritoneal mesothelioma: a case report. *Journal of Medical Case Reports*.

[11] Sethna K, Mohamed F, Marchettini P, Elias D, Sugarbaker PH (2003). Peritoneal cystic mesothelioma: a case series. *Tumori*.

[12] Soreide JA, Soreide K, Korner H (2006). Benign peritoneal cystic mesothelioma. *World Journal of Surgery*.

[13] Muscarella P, Cowgill S, DeRenne LA, Ellison EC (2004). Retroperitoneal benign cystic peritoneal mesothelioma. *Surgery*.

